# *In vitro*, but not *in vivo*, reversibility of peritoneal macrophages activation during experimental acute pancreatitis

**DOI:** 10.1186/1471-2172-10-42

**Published:** 2009-07-31

**Authors:** Sabrina Gea-Sorlí, Daniel Closa

**Affiliations:** 1 Dept. Experimental Pathology, IIBB-CSIC-IDIBAPS-CIBEREHD, Barcelona, Spain

## Abstract

**Background:**

Systemic inflammatory response syndrome is one of the major pathobiologic processes underlying severe acute pancreatitis and the degree of macrophage activation could be one of the factors that finally determine the severity of the disease. We evaluated the activation phenotype in peritoneal macrophages during the progression of an experimental model of acute pancreatitis induced in rats by intraductal administration of 5% sodium taurocholate and the effect of IL-4 and IL-13 to modulate this activation.

Samples of pancreas, lung and adipose tissue as well as plasma were also obtained. In some animals IL4 and IL13 were injected 1 h after induction in order to modulate macrophage activation. The expressions of TNFα and Mannose Receptor, as indicators of classical and alternative macrophage activation, were evaluated. Levels of myeloperoxidase and plasma lipase were determined to evaluate the severity of the inflammatory process. The stability of IL-4 in ascitic fluid and plasma was evaluated.

**Results:**

Peritoneal macrophages showed a classical M1 activation clearly induced 3 h after pancreatitis induction and maintained until 18 h. Treatment with IL-4 and IL-13 reversed the activation of macrophages from a classical M1 to alternative M2 *in vitro*, but failed to modulate the response of peritoneal macrophages *in vivo *despite a reduction in inflammation was observed in lung and adipose tissue. Finally, IL-4 shows a short half-live in ascitic fluid when compared with plasma.

**Conclusion:**

Peritoneal macrophages adopt a pro-inflammatory activation early during acute pancreatitis. Treatment with M2 cytokines could revert *in vitro *the pancreatitis-induced activation of macrophages but fails to modulate its activation *in vivo*. This treatment has only a moderate effect in reducing the systemic inflammation associated to acute pancreatitis. Hydrolytic enzymes presents in ascitic fluid could be involved in the degradation of cytokines, strongly reducing its utility to modulate peritoneal macrophages in pancreatitis.

## Background

Acute pancreatitis is an inflammatory process of the pancreatic gland that in the severe forms involves remote organ systems. Systemic inflammatory response syndrome (SIRS) is one of the major pathobiologic processes underlying severe acute pancreatitis. This is of major importance because half of deaths in the first weeks of the process are attributed to organ failure, and in particular the acute respiratory distress syndrome, associated with SIRS [1]. Despite advances in diagnosis and treatment of inflammatory pancreatic disease, to date, supportive care remains the only treatment for patients with pulmonary complications [2].

Several proinflammatory mediators have been identified to play a role in the progression of the local pancreatic damage to the systemic inflammation. This includes tumor necrosis factor α (TNFα), interleukin (IL)-1β, IL-6, MCP-1 or Platelet activating factor [3]. Some of these mediators are released by pancreatic acinar cells and results in the recruitment of neutrophils and monocytes. In addition, other inflammatory cell populations contribute to the systemic generation of inflammatory mediators. In particular, it has been reported that peritoneal macrophages, alveolar macrophages and Kupffer cells become activated in the early stages of severe acute pancreatitis [4-6]. Since macrophages orchestrate both the initiation and the resolution of inflammation, it is suspected that the degree of macrophage activation could be one of the factors that finally determine the severity of the process.

However, macrophages could be activated in different pathways. The initial inflammatory response is mediated by classically activated macrophages (M1) while the resolution phase is carried out by alternatively activated (M2) macrophages [7]. M1 macrophages are induced by IFNγ or LPS and synthesize and release constitutive amounts of inflammatory mediators such as TNFα, IL-1β, IL-6 and nitric oxide. The biological activities of M1 macrophages are characterized by its antimicrobial and cytotoxic properties, related with their role in host responses to infection or autoimmune diseases. By contrast, M2 macrophages, that are induced by IL-4 or IL-13, do not generate these mediators but promote proliferative and angiogenic processes [8]. These M2 macrophages play a role in modulating wound healing, suppressing the inflammatory response and synthesising extracellular matrix.

The characteristics of acute pancreatitis suggest that activation of macrophages correspond to the classical M1 phenotype. However, there are no data about the phenotypic status of different macrophage populations during the progression of acute pancreatitis and the relation between the differentiation to M1 phenotype and the severity of the disease.

In this work we have used an experimental model of acute pancreatitis induced by intraductal administration of sodium taurocholate to evaluate how the progression of pancreatitis correlates with the M1 activation of peritoneal macrophages as well as the effect of IL-4 and IL-13, administered after the induction of pancreatitis, in preventing the M1 activation and inducing the reparative M2 phenotype. We demonstrated that pancreatitis results in an M1 activation of peritoneal macrophages that could be reverted in vitro by treatment with IL-4 and IL.13. However, in vivo administration of these cytokines after induction of pancreatitis does not modulate the activation of peritoneal macrophages. The effect of pancreatitis-associated ascitic fluid (PAAF) on the cytokines could explain this lack of effects.

## Methods

### Animals

Male Wistar rats (250–300 g b.w.) were used in all experiments (Charles River, France). Animals were housed in a controlled environment and fed with standard laboratory pelleted formula (A04, Panlab, Barcelona, Spain) and tap water *ad libitum*. This study conformed to European Community for the use of experimental animals and the institutional committee of animal care and research approved it.

### Animal model of acute pancreatitis

Animals (n = 6 each group) were anesthetized with an i.p. administration of sodium pentobarbital (50 mg/kg). The biliopancreatic duct was cannulated through the duodenum and the hepatic duct was closed by a small bulldog clamp. Pancreatitis was induced by retrograde perfusion into the biliopancreatic duct of 5% sodium taurocholate (Sigma, St Louis, Missouri, USA) in a volume of 0.1 ml/100 g b.w. using a perfusion pump (Harvard Instruments, Edenbridge, UK) [9]. The same procedure was applied to control animals but receiving an intraductal perfusion of saline solution (NaCl 0.9%) instead of taurocholate. Three or eighteen hours after induction, peritoneal macrophages were obtained. Five ml of blood were obtained from the cava vein, heparinized and centrifuged to obtain plasma. Ascitic fluid, and samples of pancreas, white adipose tissue (WAT) and lung were also obtained, immediately frozen and stored at -80°C until used.

In a second experiment, IL-4 (4 μg/kg) and IL-13 (4 μg/kg) were i.p. administered to an additional group 1 h after the induction of pancreatitis. In these animals, macrophages, plasma and tissue samples were obtained three hours after the induction of pancreatitis.

### Isolation and culture of macrophages

Peritoneal macrophages were harvested by 5 peritoneal washes with 10 ml of phosphate buffered saline (PBS) containing 3 units/ml heparin. The obtained cell suspension was centrifuged (300 × g; 7 min). Cells were suspended in the RPMI1640 culture medium containing 10% fetal calf serum, 2 mM glutamine, penicillin (100 U/ml) and streptomycin (100 μg/ml). Aliquots of about 3 × 10^6 ^cells were plated in 6 wells plates and cultured at 37°C under a gas phase of air/CO_2 _(95:5). After an attachment period of 4 h, the non-adherent cells were removed by shaking. The resulting adherent population consisted of > 92% macrophages as judged by CD68 staining measured by flow cytometry.

### *In vitro *reversion of phenotypic changes in macrophages

Peritoneal macrophages obtained from control animals as well as animals 18 hours after pancreatitis induction were incubated for 24 h with IL-4/IL-13 (10 ng/ml each) in order to evaluate their capacity to reverse the acquired M1 phenotype. This mixture of cytokines has been reported to be more effective than IL-4 or IL-13 alone on inducing an M2 phenotype in macrophages [10]. After the incubation period RNA was obtained and the expression of TNFα and Mannose Receptor were evaluated by quantitative RT-PCR.

### Degradation of IL-4 in ascitic fluid and plasma

To evaluate the effect of hydrolytic enzymes presents in ascitic fluid or plasma during pancreatitis on the stability of interleukins, the half life of IL-4 on these biological fluids has been determined. Plasma or ascitic fluid obtained from animals with pancreatitis were pooled and overloaded with IL-4 (25 pg/ml). Samples were incubated at 37°C for 2 h, 1 h, 30 min and 15 min after the addition of IL-4 and the concentration of IL-4 was measured by ELISA. The experiment has been carried out by triplicate.

#### RNA isolation and RT-PCR

Total RNA from cells was extracted using the TRizol^® ^reagent (Invitrogen, Carlsbad, CA). The RNA was quantified by measurement of the absorbance at 260 and 280 nm using a NanoDrop ND-1000 spectrophotometer (NanoDrop Technologies, USA).

cDNA was synthesized using the iScript cDNA synthesis kit (Bio-Rad Laboratories, Hercules, CA), and reverse transcription was then performed on 1 μg RNA sample by adding iScript reagents. The reaction was incubated at 25°C for 5 min, 42°C for 30 min, and 85°C for 5 min, and then stored at -80°C.

Subsequent PCR amplification was performed in a DNA Engine, Peltier Thermal Cycler (Bio-Rad Laboratories, CA, USA) using IQTM SYBR Green Super mix and the correspondent rat primers: TNFα forward: 5'-AACTCCCAGAAAAGCAAGCA-3' reverse: 5'-CGAGCAGGAATGAGAAGAGG-3'; Mannose Receptor forward: 5'-GCAGGTGGTTTATGGGATGT-3' Reverse: 5'-GGGTTCAGGAGTTGTTGTGG-3'; GAPDH forward: 5'-CTGTGTCTTTCCGCTGTTTTC-3' and reverse: 5'-TGTGCTGTGCTTATGGTCTCA-3'.

Initial denaturation was followed by 40 cycles of DNA amplification with fluorescence detection at the end of the elongation step (SYBR Green format). Reactions were performed in duplicate and threshold cycle values were normalized to GAPDH gene expression. The specificity of the products was determined by melting curve analysis. The ratio of the relative expression of target genes to GAPDH was calculated by using the ΔC(t) formula.

#### Lipase

Plasma lipase was determined by using commercial turbidimetric assay kits from Randox (Antrim, U.K.), according to the supplier's specifications.

#### Myeloperoxidase

Neutrophilic infiltration was assessed by measuring myeloperoxidase (MPO) activity. MPO was measured photometrically with 3,3',5,5'-tetramethylbenzidine as a substrate. Tissue samples were homogenized with 0.5% hexadecyltrimethylammonium bromide in 50 mM phosphate buffer at pH 6.0. Homogenates were disrupted for 30 seconds using a Labsonic sonicator (Braun Biotech, Inc., Allentown, PA) at 20% power and submitted to three cycles of snap freezing in dry ice and thawing before a final 30 second sonication. Samples were incubated at 60°C for 2 hours and then spun down at 4000 g for 12 minutes. The supernatants were collected for MPO assay. Enzyme activity was assessed photometrically at 630 nm. The assay mixture consisted of 20 μl supernatant, 10 μl tetramethylbenzidine (final concentration 1.6 mM) dissolved in DMSO, and 70 μl H_2_O_2 _(final concentration 3.0 mM) diluted in 80 mM phosphate buffer, pH 5.4. The results are expressed as units (U) MPO activity per g protein.

### Statistical analysis

Data have been expressed as mean ± SEM. Means of different groups were compared using a one-way analysis of variance. Tukey's multiple comparison test was performed for evaluation of significant differences between groups. Differences were assumed to be significant when p < 0.05.

## Results

### Pancreatitis induction

**Figure 1:**
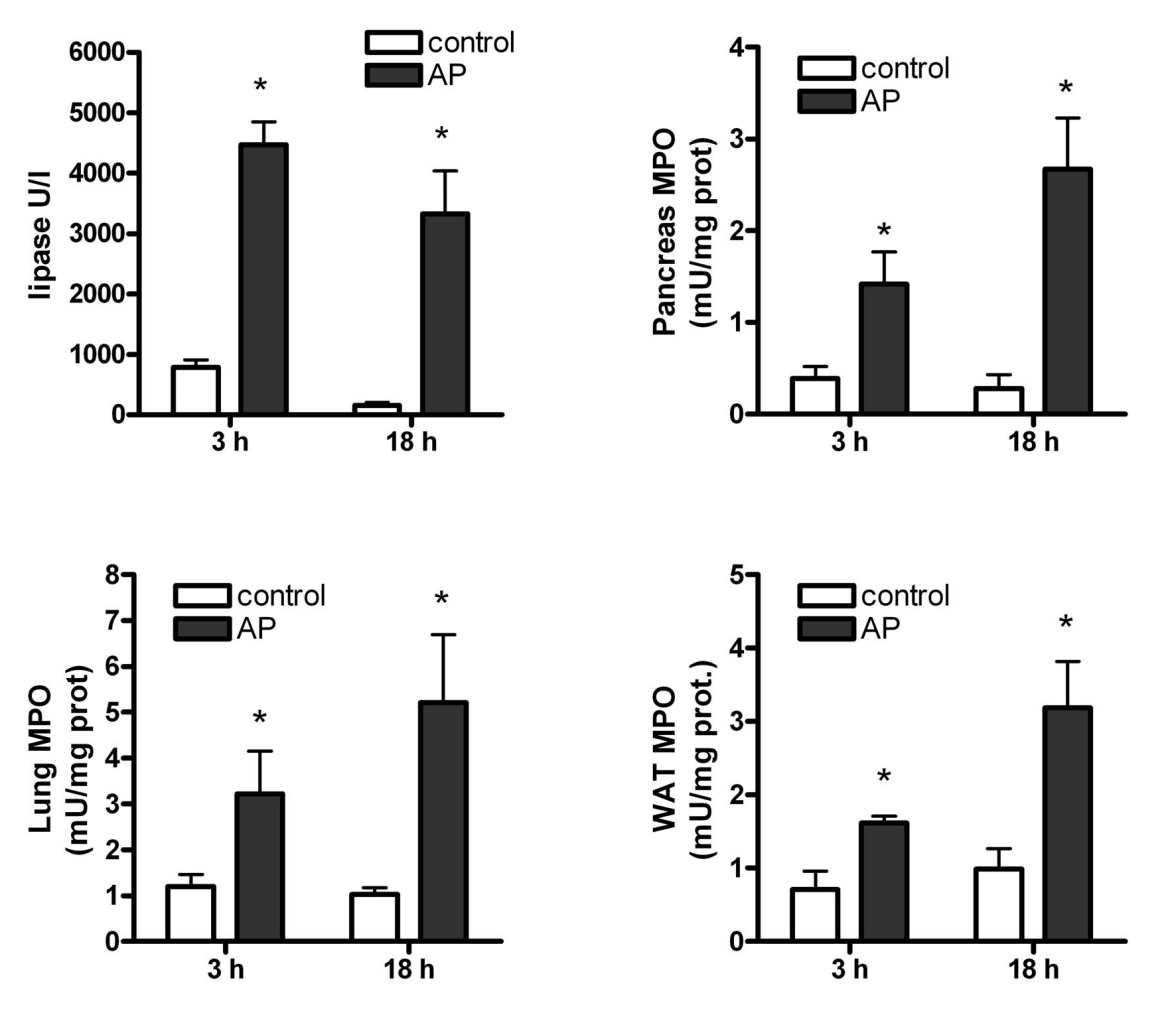
**Lipase and MPO activity in pancreas, lung and white adipose tissue (WAT) 3 and 8 hours after induction of pancreatitis**. * = p < 0.05 vs. control.

Acute pancreatitis results in a significant increase in lipase levels in plasma at 3 and 18 hours after induction. The inflammatory process, evaluated as MPO activity indicates a strong leukocyte infiltration in pancreas, WAT and lung at both time periods (figure 1).

### Macrophage activation

**Figure 2:**
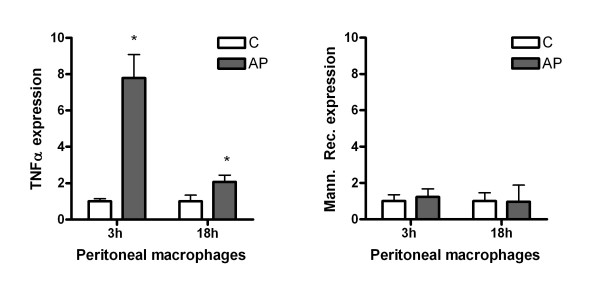
**Changes in the mRNA expression of TNFα and mannose receptor in peritoneal macrophages evaluated by RT-PCR. **Results are expressed as increases with respect to the control group. * = p < 0.05 vs. control.

Analysis of peritoneal macrophage activation indicates that 3 hours after induction it is induced a strong expression of TNFα. At this time point there was no significant changes in Mannose Receptor expression, indicating the activation following the expected M1 phenotype. Interestingly, 18 hours after induction, the expression of TNFα remains induced but in a less intense level while Mannose Receptor remained unchanged (figure 2).

### *In vitro *reversibility of the phenotype

**Figure 3:**
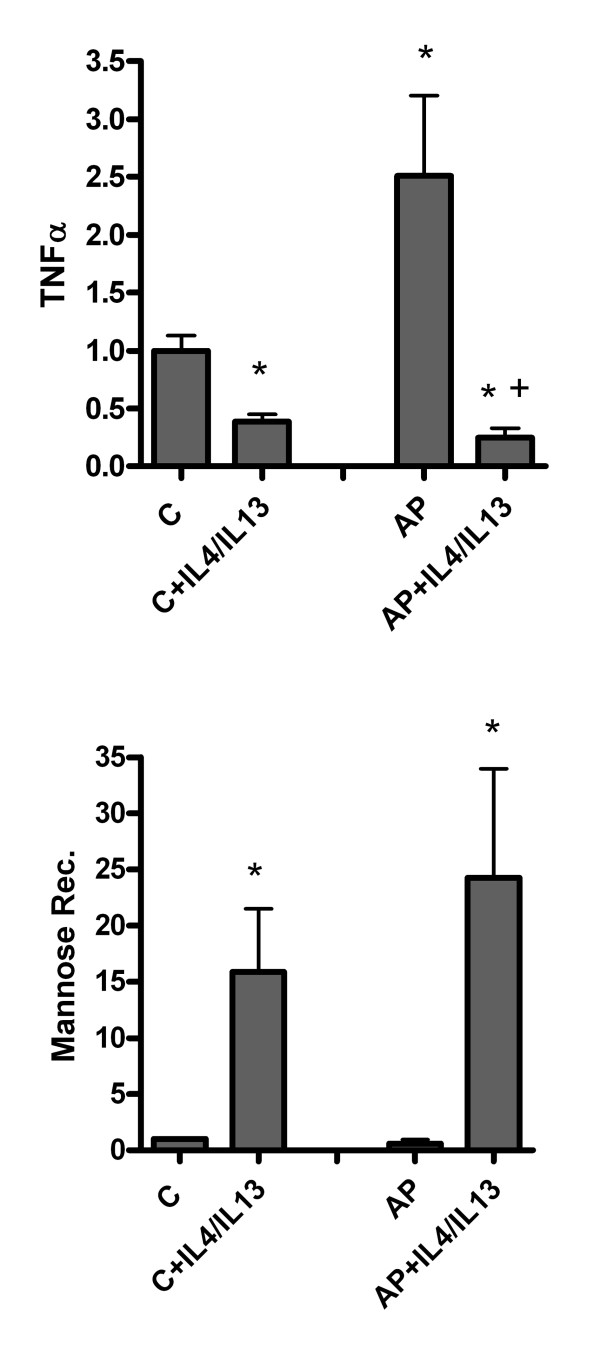
**Reversibility of macrophage phenotype was observed on incubating peritoneal macrophages obtained 18 hours after pancreatitis induction and incubated with a mixture of IL-4 plus IL-13 for 24 hours**. This treatment resulted in the inhibition of TNFα expression as well as the induction of Mannose receptor in both control and AP macrophages. Results are expressed as increases with respect to the control group. * = p < 0.05 vs. control; + = p < 0.05 vs AP.

Peritoneal M1 activated macrophages are important sources of pro-inflammatory cytokines that could contribute to the induction and maintaining of the systemic inflammatory status. Consequently in order to design therapies focussed on modulating macrophages activity it is of interest to evaluate the capacity of these cells to change their phenotype. With this purpose we treated *in vitro *peritoneal macrophages obtained 18 hours after inducing pancreatitis, with IL-4 plus IL-13, which are known to induce a M2 phenotype in macrophages [11]. Results indicate that incubation with these cytokines *in vitro *results in the reversion of the M1 phenotype and in an increased expression of Mannose Receptor, an M2 related gene (figure 3).

### *In vivo *treatment with M2 cytokines

The reversion of M1 phenotype of activated macrophages during the acute phase of pancreatitis could be an interesting therapeutical approach. With this purpose an additional group of animals was treated with IL-4 and IL-13 one hour after the induction of the disease. Two hours later (this is three hours after the induction of pancreatitis), we evaluated the progression of the inflammatory process as well as the changes in the phenotype acquired by the peritoneal macrophages.

**Figure 4:**
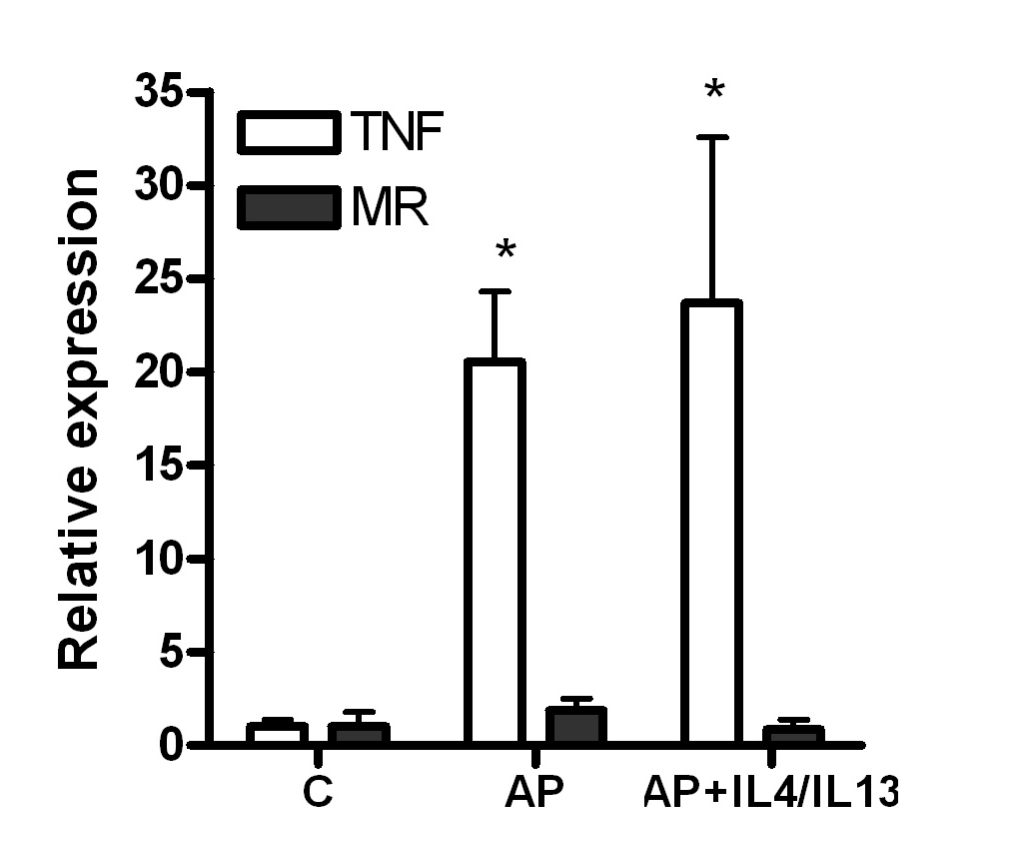
**In vivo administration of IL-4 plus IL-13 does not accomplish to reduce the macrophages activation in the early stages of pancreatitis**. No changes were observed in TNFα or Mannose receptor expression in peritoneal macrophages. Results are expressed as increases with respect to the control group. * = p < 0.05 vs. control.

**Figure 5:**
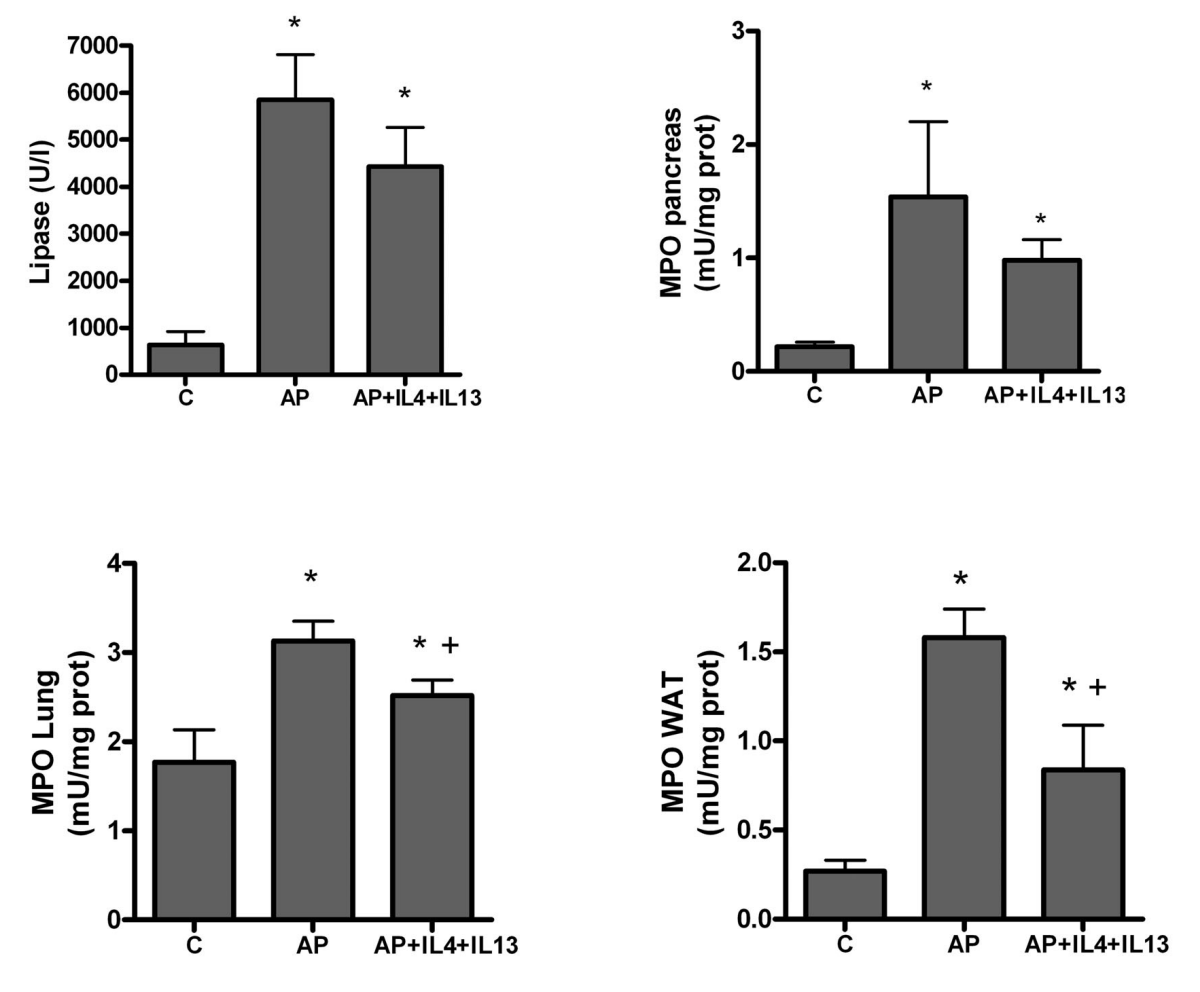
**Effect of IL-4 and IL-13 administration on the progression of the early stages of inflammation**. No changes were observed in lipase activity in plasma or in MPO levels in pancreas. By contrast, a significant inhibition of MPO in lung and white adipose tissue (WAT) indicates a reduction of the systemic inflammation. * = p < 0.05 vs. control; + = p < 0.05 vs AP.

Administration of M2 cytoquines has no effect on the M1 activation of peritoneal macrophages (figure 4). However, it results in a decrease in the inflammatory response observed in the lung and WAT (figure 5). As expected, cytokine treatment does not modify the pancreatic damage or inflammation and lipase as well as MPO in pancreas remained unchanged (figure 5).

When measuring the concentrations achieved of IL-4 in plasma no measurable levels were found even after the intraperitoneal administration of interleukins.

### Degradation of IL-4 in ascitic fluid

**Figure 6:**
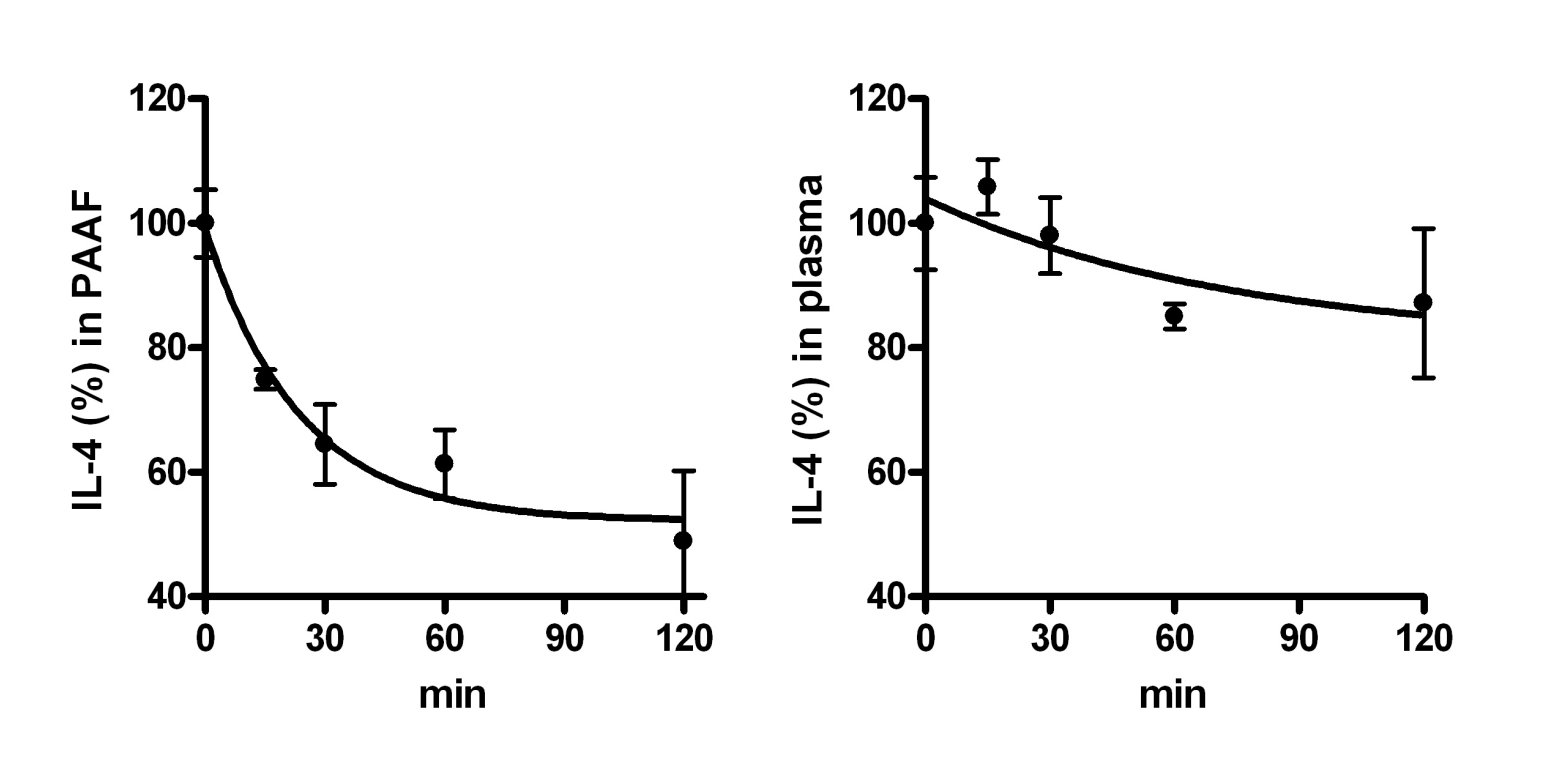
**Degradation of IL-4 in pancreatitis-associated ascitic fluid (left) and in plasma (right).** PAAF or plasma from pancreatitis were pooled and overloaded with 25 pg/ml IL-4 and the concentration was measured at different time-points. In ascitic fluid, degradation of IL-4 was observed, with a half-life of 16.4 min. By contrast, half life in plasma was 51.1 min. Values are the mean of three different experiments.

Measures of PAAF overloaded with IL-4 revealed a fast degradation of the cytokine in this biological fluid. A half life of 16.4 min was obtained, contrasting with the higher stability observed in plasma, with a half life of 51.1 min (Figure 6).

## Discussion

Systemic inflammation is a major complication of severe acute pancreatitis being the associated acute lung injury and renal failure characteristics of the end stages of the process. Measurements of cytokines in plasma of patients or in experimental models revealed increases in inflammatory mediators, including TNFα, IL-1, IL-6, MIP2, or MCP in a situation defined as "cytokine storm" that results in an uncontrolled inflammatory process in several organs [12].

In this context, macrophages play a pivotal role due to their ability to generate pro or anti-inflammatory mediators that control the progression of the inflammatory process. Different reports demonstrate the involvement of activated peritoneal, alveolar or hepatic macrophages in the pathogenesis of acute pancreatitis [5,6]. This central role in the regulation of inflammation made the macrophages interesting targets in order to design therapeutical strategies focussed in the control of the systemic effects of acute pancreatitis.

In this work we observed that peritoneal macrophages showed an early and intense M1 activation reflected in the high expression of TNFα, and the lack of changes in Mannose Receptor. This could be observed three hours after induction and remains in similar phenotypic profile at 18 h. The main difference between these two time periods is the reduction in the expression of TNFα. This fact agrees with the kinetics observed in several *in vitro *experiments when macrophages are stimulated with pro-inflammatory mediators. The initial peak of TNFα generation is followed by the expression of other cytokines, mainly IL-1β and IL-8.

This activation was expected since peritoneal macrophages are in the peritoneal cavity, in direct and early contact with mediators released by pancreatic tissue. Ascitic fluid generated in the severe acute pancreatitis contains pancreatic enzymes and cytokines in a concentration that exceeds that observed in plasma in on order of magnitude [13]. The M1 phenotype induced by these mediators can increase the inflammatory response associated with acute pancreatitis through the release of other macrophage-derived inflammatory cytokines. However, several works reported on the plasticity of activation phenotype acquired by macrophages and their capability to be reprogrammed by the effect of M1 or M2 cytokines [11,14]. Consequently, it could be of interest to known the capacity of these pancreatitis-activated peritoneal macrophages to be reverted to an antiinflammatory and reparative M2 phenotype. We have evaluated this possibility *in vitro *by treating pancreatitis-activated peritoneal macrophages with a mixture of IL-4 and IL-13. These cytokines are known to induce the alternative M2 activation of macrophages, promoting the repair phenotype and counteracting the effects of pro-inflammatory cytokines [7].

Results indicate that activated peritoneal macrophages obtained during pancreatitis could be re-directed to M2 phenotype by the effect of IL-4 and IL-13 treatment (figure 3). This was evidenced by the reduction in TNFα expression that parallels with the induction of Mannose Receptor.

In a subsequent experiment we tried to modulate *in vivo *the activation of peritoneal macrophages. For this purpose, interleukins 4 and 13 were administered i.p. one hour after the induction of pancreatitis and two hours later we evaluated the progression of the inflammatory process as well as the activation of macrophages. This was a short time period to achieve a complete M2 phenotype, but the fast progression of the disease do not allows prolonged treatments. The objective was only to achieve a reduction in the intensity of the M1 activation that could result in a decrease in the systemic inflammation.

As expected, interleukins treatment 1 h after induction has no effect on the pancreatic tissue damage which, in this model of pancreatitis, is directly induced by the effect of taurocholate on acinar cells. This was evident by the lack of changes in the increased plasma lipase activity. Pancreas inflammation also was unmodified according the MPO results. By contrast, a moderate but significant reduction in lung and WAT inflammation was observed (figure 5). However, these changes appear to be unrelated with the peritoneal macrophage activation because the M1 phenotype induced by pancreatitis remains similar to that observed in the non-treated group (figure 4).

It has been observed in several experimental models that IL-4 could modulate the regulation of complement activation [14] or the generation of IL-10 in chronic pancreatitis [15]. Some of these effects could explain the reduction in the inflammation observed in our experimental conditions. However, it is clear that interleukin administration was not enough to therapeutically modulate the M1 phenotype observed in peritoneal macrophages. This lack of changes after M2 cytokines administration could be related with the strong proinflammatory environment generated in the peritoneal cavity, but also by the hydrolytic activity of ascitic fluid.

In this sense, when measuring the stability of IL-4 in ascitic fluid we observed a fast degradation of this cytokine. These changes were not observed in plasma and could be related with the hydrolytic enzymes released by pancreatic tissue to the ascitic fluid. This fact also explains the lack of measurable increases in IL-4 levels in plasma of treated animals. It must be pointed out that the intraperitoneal administration of cytokines was selected since our objective was to act on peritoneal macrophages. Consequently, the administration of cytokines to modulate the activation of macrophages during the progression of acute pancreatitis appears to be useful for act on several cell populations but not for the peritoneal macrophages.

## Conclusion

Our results indicate that peritoneal macrophages adopt a pro-inflammatory activation early during acute pancreatitis and that they could be reprogrammed *in vitro *to a reparative M2 phenotype by IL-4 and IL-13. However, *in vivo *this treatment has only a moderate effect in reducing the systemic inflammation and fails to prevent the peritoneal macrophage activation. This lack of effect seems to be related with the degradation of interleukins by the action of ascitic fluid present in the peritoneal cavity. Consequently, different therapeutic approaches will be needed to modulate the activation of this cell population for the treatment of severe acute pancreatitis.

## Authors' contributions

SGS participated in animal surgery, carried out the *in vitro *studies and the analytical processes and helped to draft the manuscript. DC conceived of the study, and participated in its design and coordination and draft the manuscript. All authors read and approved the final manuscript.
